# Coevolution of group-living and aposematism in caterpillars: warning colouration may facilitate the evolution from group-living to solitary habits

**DOI:** 10.1186/s12862-020-01738-w

**Published:** 2021-02-14

**Authors:** Lingzi Wang, Stephen J. Cornell, Michael P. Speed, Kevin Arbuckle

**Affiliations:** 1 Institute of Integrative Biology, University of Liverpool, L69 7ZB Liverpool, UK; 2 School of Life Science, University of Liverpool, L69 7ZB Liverpool, UK; 3 Department of Biosciences, College of Science, Swansea University, SA2 8PP Swansea, UK

**Keywords:** Aposematic, Coevolution, Cryptic, Group, Phenotypic evolution, Solitary

## Abstract

**Background:**

Animals use diverse antipredator mechanisms, including visual signalling of aversive chemical defence (aposematism). However, the initial evolution of aposematism poses the problem that the first aposematic individuals are conspicuous to predators who have not learned the significance of the warning colouration. In one scenario, aposematism evolves in group-living species and originally persisted due to kin selection or positive frequency-dependent selection in groups. Alternatively, group-living might evolve after aposematism because grouping can amplify the warning signal. However, our current understanding of the evolutionary dynamics of these traits is limited, leaving the relative merit of these scenarios unresolved.

**Results:**

We used a phylogenetic comparative approach to estimate phenotypic evolutionary models to enable inferences regarding ancestral states and trait dynamics of grouping and aposematic colouration in a classic model system (caterpillars). We find strong support for aposematism at the root of the clade, and some (but weaker) support for ancestral solitary habits. Transition rates between aposematism and crypsis are generally higher than those between group-living and solitary-living, suggesting that colouration is more evolutionarily labile than aggregation. We also find that the transition from group-living to solitary-living states can only happen in aposematic lineage, suggesting that aposematism facilitates the evolution of solitary caterpillars, perhaps due to the additional protection offered when the benefits of grouping are lost. We also find that the high frequency of solitary, cryptic caterpillars is because this state is particularly stable, in that the transition rates moving towards this state are substantially higher than those moving away from it, favouring its accumulation in the clade over evolutionary time.

**Conclusions:**

Our results provide new insights into the coevolution of colour and aggregation in caterpillars. We find support for an aposematic caterpillar at the root of this major clade, and for the signal augmentation hypothesis as an explanation of the evolution of aposematic, group-living caterpillars. We find that colouration is more labile than aggregation behaviour, but that the combination of solitary and cryptic habits is particularly stable. Finally, our results reveal that the transitions from group-living to solitary-living could be facilitated by aposematism, providing a new link between these well-studied traits.

## Background

Animals use a rich variety of defences to protect themselves from predators. A common form of antipredator defence is protective colouration, such as cryptic or aposematic warning colouration. For example, many dart frogs (Dendrobatidae) use bright aposematic colours to warn predators of toxic chemical defence and so reduce the costs of attack, including predation [1]. Alternatively, many species have cryptic colour patterns which reduce their detectability, for instance, the green colouration of many arboreal snakes which blend in with surrounding foliage [2]. Although conspicuous colouration can function aposematically, advertising distasteful or harmful chemical defences, it can also be associated with Batesian mimicry wherein palatable individuals mimic aposematic species. Here we treat conspicuous colouration as aposematic since Batesian mimicry seems to be very rare in caterpillars [3].

Living in groups can also enable many antipredator strategies, such as the ‘dilution effect’ in which individual risk of predation decreases with increasing group size, assuming that a predator selects prey in a group randomly and can’t consume the whole group [4–7]. Moreover, aggregation can interact with protective colouration in a way which influences the costs and benefits of a given strategy. For instance, when aposematic individuals gather in a group, the combined signal may be magnified and more conspicuous (signal augmentation), so the predators are less likely to attack the prey group [8]. Indeed, the evolution of aposematism has been linked to group-living in caterpillars for many years [3], but the directional nature of the relationship remains debated. On the one hand, according to the signal augmentation hypothesis above, aposematism should evolve first and subsequently provide selection pressure to evolve grouping to enhance the warning signal [3–12]. On the other hand, kin selection [13–16] or positive frequency-dependent selection [15–17] may be important in overcoming constraints in the initial evolution of aposematism, whereby rare (new) conspicuous individuals are eaten but their kin may survive and carry genes for aposematism. Under this scenario, grouping should evolve first (for example via the presence of local kin) and subsequently facilitate the evolution of aposematism. Hence, although a link between grouping and aposematism is established, understanding the direction of this relationship can provide insights into the underlying mechanisms.

In a well-known study on caterpillars, Tullberg and Hunter [9] attempted to answer this question using early phylogenetic comparative methods and concluded that the transition to group-living is more frequent in aposematic lineages than cryptic ones. This suggests that grouping is more beneficial for aposematic than cryptic species, but their study was unable to simultaneously account for the evolutionary dynamics of both colour and aggregation traits, impairing our understanding of the relationship between these traits.

The current study revisited and expanded the work of Tullberg and Hunter [9], taking advantage of developments in comparative biology over the last two decades to address several limitations of that study. An inherent assumption in previous work was that the ancestral state for caterpillars was solitary and cryptic, but it is still possible that the caterpillar ancestor might have the group-living or aposematic state. By explicitly modelling evolutionary transitions between both colour patterns and grouping habits simultaneously and accounting for unequal transition rates we test evolutionary pathways and ancestral states more robustly than was possible for Tullberg and Hunter [9]

Additionally, phylogenetic datasets are now much more comprehensive than in the 1990s. The phylogenetic trees used by Tullberg and Hunter [9], which were analysed separately for each superfamily using disparate data sources including taxonomic classification as a proxy, were necessarily smaller than that used here, and they did not use informative branch lengths (related to time). Also, in their analyses of trait evolution, their implementation of “independent contrasts” differed from the classical independent contrasts approach described by Felsenstein [18], resulting in many branches for their defined contrasts being excluded. In contrast, here we were able to reconstruct a single phylogeny for the whole sample using a standard set of molecular data with branch lengths related to time to provide a more powerful basis for our coevolutionary analyses.

In all, we exploited advances in comparative biology and data availability to revisit Tullberg and Hunter’s [9] classic study of the coevolution of aposematism and grouping. Our approach enabled us to more robustly test their hypotheses, examine potentially important assumptions made, and expand the questions asked to gain a better understanding of the system. Specifically, we aimed to (1) investigate evolutionary transitions between combinations of grouping (vs solitary habits) and aposematic (vs cryptic) colouration to better understand their coevolutionary dynamics, and (2) estimate ancestral states to infer where these transitions occurred and in what order.

## Method

### **Trait data**

We used data on colour pattern and grouping from Tullberg and Hunter’s [9] original dataset. Colour pattern was classified as either aposematic or cryptic. Caterpillars which are strikingly marked with combinations of black and yellow, red and/or white were considered aposematic, whereas other colour patterns (such as plain green or counter-shaded) were considered to be cryptic. Grouping was classified as either group-living, where caterpillars aggregate during the whole or part of their development, or solitary if they do not aggregate. Species which lay eggs in clusters but disperse upon hatching were also treated as solitary.

The dataset we used for analyses consists of 676 lepidopteran species, of which 541 (80.0%) were coded as solitary-cryptic, 82 (12.1%) were solitary-aposematic, 21 (3.1%) were group-cryptic, and 32 (4.7%) were group-aposematic. All data used for this paper are available at https://figshare.com/s/359ff8f6c15beb68fab8.

### **Phylogenetic tree**

There were five superfamilies present in our dataset: Papilionoidea, Bombycoidea, Drepanoidea, Geometroidea and Noctuoidea (with 34, 59, 18, 300, 265 species sampled out of 676 known species respectively). Two DNA sequences, CO1 and EF-1$\alpha$, were obtained for the species in our dataset from GenBank (https://www.ncbi.nlm.nih.gov/genbank/) [19] by 1 July 2018. We were able to obtain CO1 for 667 species (98.7%), EF-1$\alpha$ for 227 species (33.6%), and both CO1 and EF-1$\alpha$ sequences for 218 species (32.2%). The accession numbers for the sequences are also available at https://figshare.com/s/359ff8f6c15beb68fab8.

We aligned the CO1 and EF-1$\alpha$ sequences using MUSCLE [20] with default settings in the software MEGA7 [21], and concatenated the aligned sequences using SequenceMatrix [22]. We used PartitionFinder2 [23], using linked branch lengths and the greedy algorithm, to estimate the best partitioning scheme and substitution model for each partition based on AICc. The best scheme consisted of four partitions, positions 1 and 2+3 in our alignment for each of the two genes, and substitution models were as follows: GTR+I+G for CO1 position 1, GTR+G for both CO1 positions 2+3 and EF-1$\alpha$ position 1, and GTR for EF-1$\alpha$ positions 2+3. This partitioning scheme was used for phylogenetic inference in BEAST v2.4 [24], and both PartitionFinder2 and BEAST were run in the CIPRES portal [25]. We linked the tree across all four partitions but allowed site and clock models to be estimated independently. We used a Yule model as the tree prior and an uncorrelated log normal relaxed clock model. Because of the limited information in two genes to estimate deep time relationships, we used a phylogenomic backbone [26] to constrain both the topology and divergence dates of deep nodes in the tree representing relationships between the superfamilies and the timing of their diversification. The underlying study used for this is the most complete phylogenomic study of lepidopterans to date, including 2098 protein-coding genes for 186 species and 16 primary fossil calibrations [26]. Based on this we constrained divergence dates of 9 nodes representing divergence events between superfamilies and their initial crown group divergence events (basal splits of each superfamily), calibrated such that prior distributions matched the 95% HPD intervals of the equivalent nodes in Figure S12 of that paper. The MCMC analysis was run for 100 million generations after a burn-in of 10 million generations, and samples were stored every 100,000 generations, resulting in a posterior distribution of 1001 trees. The maximum clade credibility tree was calculated from this distribution in the phangorn package v2.5.3 [27] in R v3.5.1 [30], and this was used for all comparative analyses herein.

### **Analysis of the coevolution of colour and grouping**

**Fig. 1:**
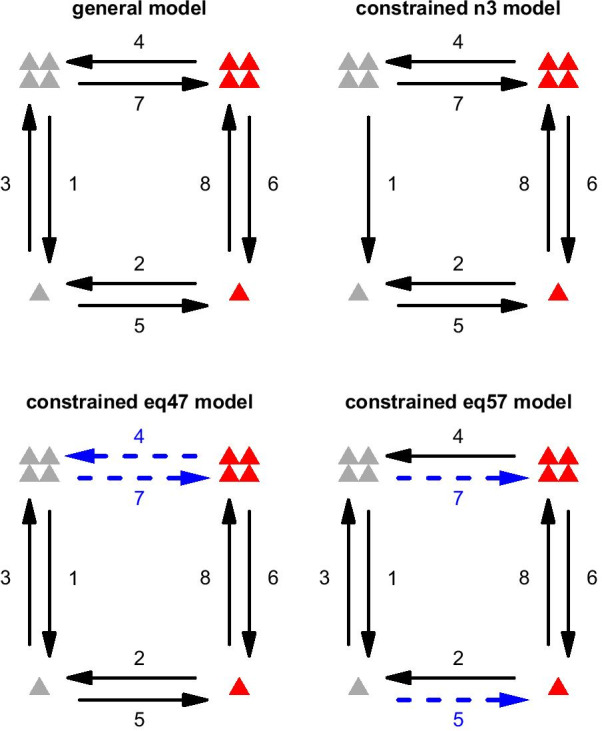
The general model and the constrained models. Diagrammatic representations of the general model with no constrained transition rates; an example of a constrained zero model (n3 model) with some transition rates (in this case rate 3, from cryptic solitary to cryptic group) constrained to 0, and examples of two types of constrained equal models (eq47 model and eq57 model) with two of the transition rates (shown as blue dashed arrows) equal. Figure symbols: cryptic (grey), aposematic (red); solitary-living (one triangle), group-living (four triangles)

We estimated evolutionary pathway models between each of the four states combining the binary traits of aposematism and grouping following Pagel’s [28] method for estimating transition rates (Fig. 1). Pathway models were estimated via maximum likelihood using the function corDISC in the package corHMM 1.22 [29] implemented in R 3.5.1 [30].

We first estimated transition rates for a general model which has no constraints (Fig. 1 top left). To explicitly test for alternative evolutionary pathways we also fitted a series of constrained models in which different combinations of the transition rates were constrained to equal 0. Figure 1 (top right) shows one example of this set of constrained models in which transition rate 3 (cryptic solitary $\rightarrow$ cryptic group) was assumed to be impossible and constrained to be 0. We had named our models such that the unconstrained model was called ‘general’ and the various models with rates constrained to 0 were named as in the following examples. We used ‘n3 model’ for the constrained model with the transition 3 not possible, shown in Fig. 1. Similarly, a model with both rates 3 and 4 set to 0 was referred to as ‘n34 model’. We exhausted all the possible constrained zero models, giving $2^8-2=254$ models in total with this type of constraint.

Furthermore, we tested a different set of constrained models in which two transition rates were constrained to be equal to each other. Specifically, we tested (1) models with symmetrical transition rates, e.g. where the transition rate from group-living aposematic to group-living cryptic is equal to that from group-living cryptic to group-living aposematic (Fig. 1 bottom left), and (2) models where the transition rates from one state to the other for a given trait are independent of the state of the other trait, for instance the rate of evolution from crypsis to aposematism in group-living caterpillars is equal to that for solitary caterpillars (Fig. 1 bottom right). The corresponding models were named after the transition rates which were equal (e.g. eq47 model was named for the constrained model with the transition rates 4 and 7 equal). There were 4 models of type (1): eq47 model, eq68 model, eq25 model, eq13 model, and 4 models of type (2): eq57 model, eq24 model, eq16 model, eq38 model.

We then compare the evidence for each of our models using an information theoretic approach based on Akaike’s information criterion (AIC).

We estimated ancestral states across our phylogeny using the best fitting pathway model in a maximum likelihood framework, implemented using the plotRECON function in the R package corHMM [29]. Incorporating our pathway modelling results into our ancestral state estimation should improve performance by accounting for any inferred constraints on the evolution of the traits, basing them on the best supported model of trait evolution.

**Table 1:** The ten best models according to Akaike’s Information Criteria (AIC)

	K	logLik	AIC	$\Delta$AIC	LikRatio	AkaikeWeight
n1	7	$-$362.705	739.410	0.000	1.000	0.201
eq24	7	$-$362.754	739.508	0.099	0.952	0.192
eq13	7	$-$362.765	739.530	0.120	0.942	0.190
eq18	6	$-$364.216	740.431	1.022	0.600	0.121
General	8	$-$362.705	741.410	2.000	0.368	0.074
eq38	7	$-$363.804	741.609	2.199	0.333	0.067
eq16	7	$-$364.034	742.068	2.659	0.265	0.053
n8	7	$-$364.216	742.431	3.022	0.221	0.044
eq47	7	$-$364.674	743.347	3.938	0.140	0.028
eq57	7	$-$365.874	745.748	6.338	0.042	0.009

$\Delta$AIC= difference in AIC between each model and the best model (AIC-AIC$_{min}$); LikRatio = likelihood ratio between each model and the best model (exp((AIC$_{min}$-AIC)/2)), sometimes called the ’evidence ratio’ and gives the strength of evidence for each model as a proportion of the best model; AkaikeWeight = model probabilities (probablility of each model being the best model in the set) [31, 32]

## Results

### **Evolutionary pathway models**

**Fig. 2:**
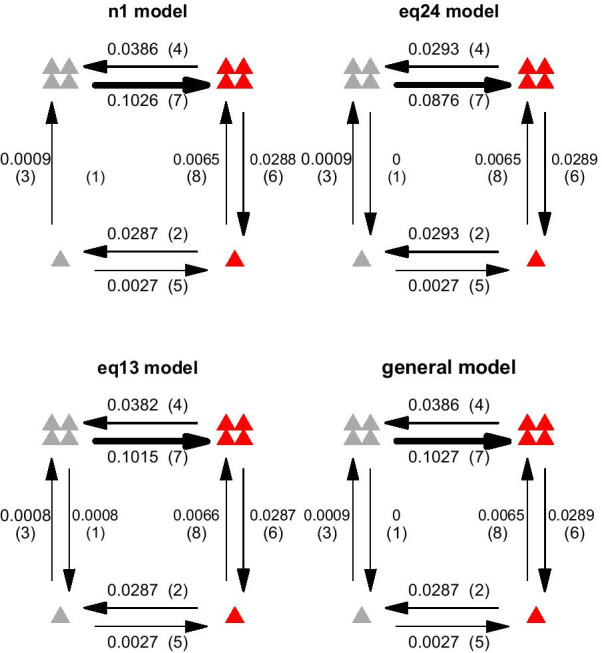
The estimated transition rates in four of the best AIC models. Four of the best AIC models (Table 1), which show the very similar parameter estimates, log-likelihoods (Table 1) and ancestral state estimates (visually the same as in Fig. 3). Figure symbols: cryptic (grey), aposematic (red); solitary-living (one triangle), group-living (four triangles). The models are named in the way shown in Fig. 1

The best ten models chosen by the AIC method are shown in Table 1, whereby the ‘best’ model is that with the lowest AIC value. The n1 model is best supported, but equivalent support exists for other models; importantly, the log-likelihoods and parameter estimates are either identical or very similar (Fig. 2), and also result in similar ancestral state estimates (visually the same so not shown separately here). Hence, despite support from model probabilities (Akaike weights) being spread across several models, the interpretation is consistent across these models, and summing the model probabilities across these highly similar models which lead to the same conclusions gives strong support for our key results.

Our best supported models reveal that the evolution of solitary habits from group-living either cannot happen in cryptic caterpillars (only aposematic ones), as in most models, or happens at a rate ${}^{\sim }35\times$ slower than in aposematic caterpillars (in the one model with a non- zero rate estimate) (Fig. 2). They also show that colouration evolves at a faster rate than aggregation tendencies, since transition rates between crypsis and aposematism are generally higher than those between solitary and group-living (Fig. 2). Finally, the combination of solitary habits and crypsis, which occurs in 80% of species in our dataset, appears to be a very stable state since transition rates towards this state are much higher than those away from it (Fig. 2).

### **Ancestral state estimation supports an aposematic ancestor**

**Fig. 3:**
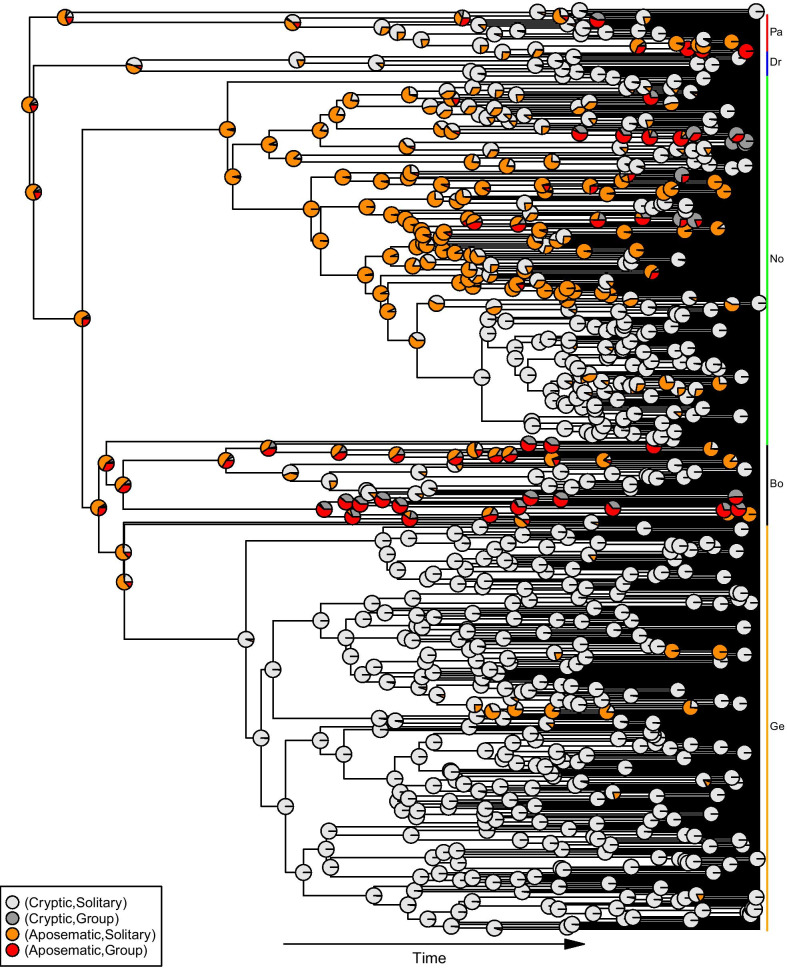
Ancestral state estimation for combinations of colour and grouping patterns. Pie charts at nodes display the relative likelihood of being in each of the four states. The colored lines and the abbreviation (Pa, Bo, No, Dr, Ge) to the right of the tips of tree mean that the corresponding tips belong to the superfamilies Papilionoidea, Bombycoidea, Noctuoidea, Drepanoidea and Geometroidea respectively

Our ancestral state estimation suggests that, contrary to previous assumptions, the ancestral caterpillar at the root of our tree was aposematic, and more likely to be solitary than group-living (Fig. 3). The subsequent evolutionary history of this clade includes several transitions to the solitary and cryptic state, which persists for long periods and characterises 80% of the species in our dataset. Several of these transitions occurred in species-rich subclades which, in combination with the evolutionary stability of this state in our transition rate models, accounts for the high frequency of the strategy.

## Discussion

### Solitary cryptic is likely to be the most stable derived state and solitary aposematic is likely to be the ancestral state for caterpillars

This research was initiated from the debate about whether the evolution from crypsis to aposematism is typically before or after the evolution from solitary to group-living. The hypothesised pathway via kin selection or positive frequency-dependent selection [13–17] predicts that a transition from solitary to group-living comes first, and then facilitates the evolution of conspicuous warning colouration. An alternative pathway, via signal augmentation [3–12] predicts that the evolution of aposematism precedes the evolution of group-living since group enhances the effect of aposematism. Our results challenge the underlying premise of both of these hypotheses and instead find strong evidence for an aposematic ancestral state (leaving the subsequent evolution of crypsis as requiring explanation). Nevertheless, our analyses suggest it is most likely that the ancestral state in this group is solitary-aposematic, suggesting that the transition of historical interest in this group (solitary-cryptic to grouping-aposematic) most likely occurred via the evolution of aposematism first. Our pathway models support this interpretation in that the rate from solitary-cryptic to solitary-aposematic is 2-3$\times$ higher than that to grouping-cryptic, favouring the signal augmentation hypothesis.

The coevolution dynamics (Fig. 2) is more likely to go in a clockwise direction since the transition rates in the clockwise direction (rates 7, 6, 2, 3 in Fig. 2) are all higher than the corresponding transition rates in the counterclockwise direction(rates 4, 8, 5, 1 in Fig. 2). The comparatively lower transition rates between the group-cryptic and solitary-cryptic states than the other transition rates, means that the transition is less likely to happen between these two states. Therefore, the coevolutionary dynamics lead to greater stability at the solitary-cryptic state. Furthermore, because we find that colouration is more evolutionarily labile than grouping, and the ancestral state for this clade is most likely solitary-aposematic, the evolution of solitary-cryptic caterpillars should be relatively easy to occur. We propose that these attributes of the coevolutionary dynamics between the two traits explain the high frequency of solitary-cryptic caterpillars in our datasets and in nature, and might also explain why previous research focused on the understanding of coevolution between colour and aggregation state from an assumed solitary-cryptic ancestor.

### Coevolution between colour and aggregation states: aposematism might facilitate the transition from group-living to solitary-living

The most likely transition from the inferred solitary-aposematic ancestor of the clade is towards a solitary-cryptic state but, importantly, it is still possible for the former to evolve group-living. Considering the evolution of aggregation strategies within aposematic caterpillars, the rate of evolution of solitary habits is an order of magnitude higher than that for the evolution of group-living. This may reflect costs to group-living in aposematic caterpillars such as increased predation by toxin-resistant predators due to the greater conspicuousness of groups [7], perhaps explaining why aposematic caterpillars are ${}^{\sim } 2.5\times$ more likely to be solitary (despite the previous focus on group-living aposematic species). In the context of evolution of aggregation strategies, an interesting and strongly supported result from our analyses is that transitions from group-living to solitary-living can only happen in aposematic species, not cryptic species. Indeed, the only model with reasonable support that disagreed on this point still found that solitary habits evolved from grouping ${}^{\sim } 35\times$ faster in aposematic caterpillars than cryptic ones. This suggests that aposematism may facilitate the evolution of solitary habits, even if those solitary species are then ultimately more likely to evolve cryptic colouration. Although less dramatic than for transitions to solitary habits, aposematism is also associated with faster rates of evolution from solitary habits to group-living, and hence generally for aggregation tendencies. We suggest that aposematism provides an additional level of protection above that conferred by group benefits, hence loosening evolutionary constraints against changes in aggregation status. Importantly, our finding that the evolution of grouping is more likely in aposematic than in cryptic species agrees with Tullberg and Hunter’s [9] results, which they interpreted as support for the signal augmentation hypothesis (just as we do with our more direct comparison above). Our ability to recover the results of Tullberg and Hunter’s original work [9] using appropriate comparisons in our study demonstrates a congruence that adds weight to our more powerful approach and the insights provided.

In solitary caterpillars, the transition rate from aposematic to cryptic colouration is an order of magnitude higher than the rate from cryptic to aposematic colouration. This may indicate that staying cryptic is more beneficial than warning predators for solitary individuals, perhaps because of the increased chance of being spotted and consumed (without group benefits) in conspicuous singletons. This explanation is consistent with a kin selected (or similar) origin of aposematism since it suggests costs to being aposematic when solitary, as does faster evolution of aposematism than crypsis in group-living caterpillars [13–17]. These results explain the tight relationship between group-living and aposematism since group-living relatively rapidly leads to aposematism and hence limits the opportunity to observe cryptic group-living caterpillars, the rarest state in our dataset (3.1% of species). Nevertheless, a holistic interpretation of our pathway models suggests that signal augmentation is the more common route to group-living aposematic caterpillars, even if alternative scenarios are possible.

The net result of these transition rates is that colour is relatively more evolutionarily labile than aggregation, and the dynamics of the system are primed to generate a large proportion of the relatively stable solitary cryptic state. The loss of group-living might be facilitated by the protection of warning colours and its related aversive chemical defence. Furthermore, the transition rates between group states (group/solitary) are higher in the aposematic species than in the cryptic species, and the transition rate between two colour states (aposematism/crypsis) is higher in group states than in solitary states. This is probably driven by the synergistic effects of aposematism and group-living in terms of increasing conspicuousness, and vice versa for crypsis and solitary-living. This result agrees with Tullberg, Leimar and Gamberale-Stille [10] who found no difference in attack rates by naive predators on cryptic and aposematic prey in groups, but the attack rate on the aposematic prey is significantly lower than on the cryptic prey in solitary individuals. It is also consistent with Alatalo and Mappes [15] who showed that the relative mortality caused by naive predators was more similar between group aposematic and group cryptic unpalatable prey than between solitary aposematic and solitary cryptic unpalatable prey.

### Implications, future work and limitations

The observed pattern of high frequencies of solitary cryptic caterpillars (combined with less informative comparative methods) may be why previous research focused on understanding the evolution of aposematic group-living animals from a solitary cryptic ancestor ([3–17]). Hence, future work to understand the loss of group-living and so the evolution of solitary habits, may prove fruitful. Since we find that the evolution from group-living to solitary-living can likely only happen in aposematic lineages, we specifically encourage future work to understand how aposematism facilitates the loss of group-living. We suggest one possibility is that being solitary is relatively risky in cryptic lineages and so reducing predation risk with warning signals facilitates the loss of group-living by compensating the added risk with another defence which deters attacks. Alternatively, group-living may not increase conspicuousness by as great a magnitude in cryptic species compared to aposematic species, such that selection for solitary habits is weaker in cryptic lineages. Under this scenario, other benefits of group-living may prevent its loss in cryptic species, whereas the balance of costs and benefits of group-living in aposematic species may be more similar to those of solitary life. In any case, our results provide new insights into the coevolution of protective colouration and grouping tendencies in a long-standing model system, and in doing so show the benefit of revisiting previous studies in ecology and evolution using newer and more powerful methodological approaches.

Caterpillars have many advantages for studies such as ours, hence their frequent use as model systems for antipredator mechanisms. However, Macrolepidoptera is a very large clade containing over 90,000 described species, which limits the feasibility of a high proportion of taxon coverage. Therefore one limitation to our research is that our analysis is based on the dataset of Tullberg and Hunter [9] and we assumed that the dataset is (1) reasonably unbiased in sampling species with respect to their traits, and (2) sufficiently informative to draw general conclusions about the coevolutionary dynamics of the group and colour states. It is possible that these assumptions do not hold and that the dataset is biased in a way that misrepresents the real underlying patterns. Nevertheless, other studies [33–36] have also shown the similar frequency distribution of colouration and aggregation in caterpillars, and we suggest that our data are unlikely to be biased in a consistent way across the five superfamilies included herein given their different lifestyles and general ecology. Hence, we believe our dataset is sufficiently representative to draw meaningful conclusions.

## Conclusion

This research revisits the debate about the evolutionary order between aposematism and group-living. Our results suggest that (1) ancestral caterpillars for our clade were aposematic and probably solitary, (2) protective colouration strategies are more evolutionarily labile than aggregation tendencies, (3) solitary, cryptic caterpillars are a relatively stable state which explains their high frequency, and (4) solitary habits exclusively (or nearly so) evolve in aposematic lineages. We propose that aposematism acts as a facilitator of the evolution of solitary from grouping habits, and that signal augmentation is the most common route to the evolution of aposematic group-living caterpillars. Our results also provide new avenues for future research focused on how aposematism, and perhaps other chemical defences, might facilitate the evolution from group-living to solitary-living in animals.

## Data Availability

The datasets generated and analysed during the current study are available at the website https://figshare.com/s/359ff8f6c15beb68fab8.
